# Socioeconomic Inequalities in the Risk Factors of Noncommunicable Diseases Among Women of Reproductive Age in Sub-saharan Africa: A Multi-Country Analysis of Survey Data

**DOI:** 10.3389/fpubh.2018.00307

**Published:** 2018-10-24

**Authors:** Sanni Yaya, Olalekan A. Uthman, Michael Ekholuenetale, Ghose Bishwajit

**Affiliations:** ^1^School of International Development and Global Studies, University of Ottawa, Ottawa, ON, Canada; ^2^Division of Health Sciences, Warwick Centre for Applied Health Research and Delivery, Warwick Medical School, University of Warwick, Coventry, United Kingdom; ^3^Department of Epidemiology and Medical Statistics, Faculty of Public Health, College of Medicine, University of Ibadan, Ibadan, Nigeria

**Keywords:** high blood pressure, obesity, tobacco, concentration index, Lorenz, global health, sub-Saharan Africa, women's health

## Abstract

**Background:** Understanding the socioeconomic discordance associated with the risk factors of non-communicable diseases (NCDs) can help direct effective interventions to end its persistent occurrence. We examined the prevalence of high blood pressure, overweight/obesity, alcohol consumption and tobacco use among women and compared across wealth quintiles in sub-Saharan Africa countries.

**Methods:** This study included 454,080 women of reproductive age (15–49 years) from the current Demographic and Health Survey (DHS) conducted between 2008/09-2017 across 33 sub-Saharan Africa countries. The outcome variables were high blood pressure, overweight/obesity, alcohol consumption and tobacco use. The prevalence of the risk factors of NCDs and sample characteristics across different levels of wealth quintiles were examined. Furthermore, socioeconomic inequalities were measured using concentration index (CI) and Lorenz curve considering urban-rural differentials.

**Results:** The prevalence of high blood pressure and overweight/obesity were 1.2–17.3% and 6.7–44.5% respectively with significant wealth quintile differences. More so, alcohol consumption prevalence was 4.1–47.3% and tobacco use was 0.3–9.9%. The overall prevalence of high blood pressure was 5.5%, overweight/obesity accounted for about 23.1%, alcohol consumption and tobacco users were 23.9 and 2.4%, respectively. The socioeconomic inequalities in high blood pressure (CI = 0.1352, *p* < 0.001); overweight/obesity (CI = 0.2285, *p* < 0.001), and alcohol consumption (CI = 0.0278, *p* < 0.001) were significantly more in the higher socioeconomic group, compared to the lower socioeconomic group. In contrast, the prevalence of tobacco use (*CI* = −0.2551, *p* < 0.001) was significantly more in the lower socioeconomic group, compared to the higher socioeconomic group. The test for differences in rural vs. urban concentration indices for high blood pressure, overweight/obesity, alcohol consumption, and tobacco use were statistically significant in all the health indicators (*p* < 0.05).

**Conclusion:** An effective intervention should incorporate a high-risk approach to terminate risk distribution by directing resources to key population women. To improve the benefit to risk ratio and enhance the cost effectiveness of preventive health programmes, it is paramount to understand the worth of equity-based strategies. Integrating equity elements to interventions is a key measure toward ensuring that policies and programmes meet their milestones. Government should strengthen living standards, literacy and healthcare system to curtail the increasing prevalence of the risk factors of NCDs.

## Background

Inequalities in health outcomes were a major driver of the Sustainable Development Goals (SDGs) which characterize global efforts toward universal health coverage, particularly SDG-3. The point of the goal is to help ensure healthy lives and promote well-being for all at all ages (1). SDGs also target reducing premature mortality from non-communicable diseases (NCDs) by about one-third by 2030 (1). Notably, NCDs are gradually overriding health care needs in resource-constrained settings and gaining in-depth policy recognitions. While about 50% of premature NCD mortality is reported in low- and middle-income countries (LMICs), the problem of NCDs is increasing rapidly with socio-economic discordance (2). NCDs share notable behavioral risk factors; including unhealthy diet, increased alcohol consumption, tobacco use, and sedentary lifestyles, which consequently lead to other metabolic risk factors including overweight and obesity, increased level of blood pressure, increased level of blood glucose, and high level of cholesterol (2). These continue to pose large public health problems in several developing countries.

Researchers have reported that hypertension, diabetes, tobacco use, dyslipidemia, overweight, and obesity are prominent risk factors of NCDs (3). Considering the differences in outcome and exposure indicators, the burden of behavioral risk factors of NCDs is influenced by socio-economic characteristics within resource-constrained settings (4). Socio-economic characteristics have been identified as a major driver in the distribution of NCD risk factors (5), which has led to a growing concern in the measurement of inequality in health care (6). Besides, inadequate resources, an aging population and a deficient health system also present substantial hindrances to end the burden of NCDs (5). Overall interest in socioeconomic inequalities in health also extends beyond measurement through to understanding and explaining its underlying causes.

Despite efforts to improve lives and well-being, the burden of hypertension and other metabolic risk factors of NCDs still persist, with large segments of the population in Africa affected (7).

Hypertension is a leading risk factor for cardiovascular illnesses and affects approximately 1 billion people globally (8). It is one of the prevalent determinants for cardiovascular illnesses in most rural and urban communities in sub-Saharan Africa and contributes to the increasing burden of cardiovascular diseases with little control which is quite alarming (9). Worst still, many people who have hypertension are ignorant of their health condition and even among those with confirmed hypertension, skilled care or compliance to treatment is usually lacking (10). Hypertension is a critical public health issue especially in economically developing countries. Further, cardiovascular diseases have also become the prominent cause of death globally and also a key public health problem in many resource-constrained settings (10, 11).

In addition, overweight and obesity are global health problems, specifically among women in urban communities. Their increase among women of reproductive age have recently doubled in many African countries (12). The consequences of overweight and obesity on women are critical, particularly during pregnancy which is accompanied with numerous adverse outcomes (13, 14). Obesity is a risk factor for numerous NCDs including cardiovascular diseases, stroke, and various forms of cancer resulting to illness and premature death (15). The recent shift in nutritional patterns has posed public health problems such as increase in the prevalence of overweight and obesity (16). There are disparities in the prevalence of overweight and obesity across countries and socio-economic levels (17). The prevalence of obesity across 32 Sub-Saharan African Countries ranged from 1.1% in Madagascar to 23.0% in Swaziland. More so, women in urban communities or with higher socioeconomic status were more likely to be overweight and obese, than the rural dwellers and those with lower socioeconomic status (17), Over the past few decades, the burden of obesity has increased in LMICs and no substantial reduction has been reported in developed countries (18, 19).

Tobacco use is a behavioral risk factor for NCDs that has adverse effects on the body ranging from respiratory and cardiovascular diseases to reproductive problems (20). The World Health Organization (WHO) has reported up to a billion people use tobacco worldwide. Unfortunately, tobacco use comes with an excruciating public health cost of an early death of almost 50% of its users (21, 22). In the next three decades, the global tobacco epidemic may kill approximately 10 million people annually with about 70% of these deaths occurring in LMICs (23). In spite of the overall reduction in cigarette smoking, the consumption of alternative forms of tobacco products has risen in many countries. Chewing tobacco and waterpipe smoking are two prominent alternative forms of tobacco use in many populations (24). Lower socio-economic profile and advanced age are notable determinants of maternal use of chewing tobacco and waterpipe smoking (24). Tobacco use is known to be associated with adverse perinatal outcomes. Maternal smoking and second-hand environmental tobacco exposure, have been associated with increased risks of infants being born with congenital anomalies, low birth weight, and trends toward smaller head circumferences (25).

Alcohol use was one of the foremost determinants for global disease burden in 2010 (26). Globally, the impact of various risk factors to disease burden has been altered greatly, with a transition from risks for NCDs in childhood toward those for NCDs in adults. These variations are connected to the aging population, reduced mortality among under-five children, transition in cause-of-death composition, and in exposures to risk factor. The consumption of alcohol has been linked with increased risks for several acute and chronic diseases (27). Other methods of alcohol consumption can be dangerous and vary notably among and within different communities, and by socioeconomic class (27). The occurrence of diseases and their risk factors across communities has been reported, but there is paucity of data for the socioeconomic distribution of risk factors across LMICs. The findings from previous research revealed high burden of selected NCDs among the low wealth quintile populations in rural areas and wealthy populations in urban areas (5). In this study, we hypothesize that there is a significant difference in the distribution of NCDs risk factors across socio-economic status. Therefore, we examined socio-economic inequality of the metabolic and behavioral risk factors of NCDs.

## Methods

### Data source

This study included 454,080 women aged 15–49 years from current DHS conducted between 2008/09-2017 across 33 sub-Saharan Africa countries. DHS is a major source for the provision and monitoring of vital statistics as well as population health indicators. DHS collects a wide range of information with the target on indicators of reproductive health and fertility, maternal and child health, nutrition, mortality, and health-seeking behaviors or lifestyles (28). DHS data are useful in public health research in monitoring of prevalence, rates, trends, and inequalities. During the survey, a multi-stage stratified cluster sampling approach was used to select the respondents based on allocation of specific numbers of clusters to urban and rural settlements in the country. Different questionnaires were designed to obtain information related to women, men, households, children, and couples. The reliability and validity of the questionnaires were well conducted using standard methods. DHS has used several mechanisms to ensure high quality of data collected by avoiding sampling errors. The careful selection and training of field workers or interviewers is crucial since the data collection process include collecting biological data, such as height, weight, and blood samples. Furthermore, DHS matches interviewers with respondents based on gender: numerous questions asked in the DHS are of a sensitive or personal nature, and respondents are likely to feel more comfortable sharing this kind of information with someone of the same sex. Therefore, men interview men, and women interview women. An overview of the DHS along with an introduction to the potential scope for these data are reported elsewhere (29). DHS datasets are available for researchers online (http://dhsprogram.com/data/available-datasets.cfm).

### Variables selection and measurement

#### Outcome variable

We extracted, four risk factors of NCDs including high blood pressure, overweight/obesity, alcohol consumption and tobacco use. These factors were assessed using standard methods, as previously described (30). Blood pressure was measured using a Life Source UA-767 Plus blood pressure monitor (A&D Medical, San Jose, USA), as recommended by the World Health Organization (WHO). Three measurements were taken at approximately 10-min intervals and the respondent's blood pressure was obtained by averaging the measurements. High blood pressure was defined as systolic blood pressure (SBP) ≥140 mmHg and/or diastolic blood pressure (DBP) ≥90 mmHg. Body mass index was based on height and weight and was defined as weight in kilograms divided by the square of height in meters. Criterion variables were constructed on the basis of the WHO categories, except that small frequencies necessitated combining the underweight and normal weight (≤24.9 kg/m^2^) and overweight/obesity (≥25 kg/m^2^) (31). Furthermore, alcohol consumption was measured in binary form (yes/no) using the question; “Consumption of alcoholic drink.” For tobacco product use, women were asked questions about whether, at current, they smoke cigarettes, pipes, chews tobacco, snuffs by nose, snuffs by mouth, smokes kreteks, smokes cigars/cheroots/cigarillos, smokes water pipe, smokes other country-specific tobacco products, does not use cigarettes or tobacco. Based on the response, each woman was classified as tobacco product user vs. non-user.

#### Explanatory variables

The primary explanatory variable was wealth-related quintile. A list of household assets including floor types; roof and wall materials; access to sanitation and potable water; type of cooking fuel; ownership of radio; television; bicycle; motorcycle; refrigerator amongst others were used to measure wealth scores using principal components analysis (PCA) approach. These items are available in all DHS surveys. Based on DHS analysis of household assets, using household assets, PCA provides plausible and defensible weights for an index of assets to serve as a proxy for household wealth status. By definition, the first principal component variable across individuals or households has a mean of zero and a variance of λ, which corresponds to the largest eigenvalue of the correlation matrix of *x*. The first principal component *y* yields a wealth index that assigns a larger weight to assets that vary the most across households so that an asset found in all households is given a weight of zero (32). Weights (effectively defined by factor scores) for each asset were computed (33). Then, a relative wealth variable was created in the dataset. Thus, the wealth index takes into account the distribution of assets in order to reflect each household's economic conditions. Based on the weighted wealth scores, households were grouped into wealth quintiles; poorest (lowest level), poorer, middle, richer, and richest (highest level) (34). Other explanatory variables include age: 15–19/20–24/25–29/30–34/35–39/40–44/45–49; place of residence: urban/rural; religion: Christianity/Islam/others; education: no formal education/primary/secondary/higher; currently working: yes/no; marital status: never in union/currently in union or living with a man/formerly in union/living with a man; parity: nulliparous/1–3/ ≥ 4.

### Ethical consideration

Secondary data from the current DHS were analyzed. The DHS obtained ethical clearance from the ethical committees of the respective countries prior to the commencement of the surveys. In addition, written informed consent was usually obtained from all respondents before participation. All DHS are approved by Inner City Fund (ICF) International and Institutional Review Boards (IRB) to determine the protocols are in compliance with the United States (U.S.) Department of Health and Human Services regulations for the protection of human subjects. The data were completely anonymized and the study did not require further ethical clearance.

### Data analysis plan

Data representation was adjusted for in all analyses to account for sample weight, stratification and clustering. The prevalence of the risk factors of NCDs and sample characteristics across different levels of wealth quintiles were examined using descriptive analysis. Lorenz curves and concentration index were used to examine socioeconomic inequalities for health outcomes (35, 36). Lorenz curves were used to present socioeconomic inequalities as a plot of cumulative proportion of health indicator among women against cumulative proportion of the population ordered by wealth index. The Concentration Index (CI) is positive when the Lorenz curve is below the line of equality indicating the concentration of health variable concentrates among high socioeconomic groups and *vice versa*. The urban vs. rural place of residence was used for stratified analyses. In the Lorenz curves, individuals were ranked according to ascending wealth-related status to estimate their position in the cumulative distribution of socioeconomic status. Statistical significance was determined at *p* < 0.05. Data analysis was conducted using STATA Version 14 (STATA Corp., College Station, TX, USA).

## Results

Results showed that the prevalence of metabolic risk factors of NCDs including high blood pressure and overweight/obesity were 1.2–17.3% and 6.7–44.5%, respectively. Furthermore, for behavioral risk factors; alcohol consumption was 4.1–47.3% and tobacco use was 0.3–9.9%. See Table 1 for details.

**Table 1:** Distribution of metabolic and behavioral risk factors of NCDs among women in sub-Saharan African countries; Demographic and Health Surveys, 2008–2017.

**Country**	**Year**	**Sample size (*n*)**	**High blood pressure (%)**	**Overweight/obesity (%)**	**Alcohol consumption (%)**	**Tobacco use (%)**
Benin	2012	16,599	4.3	26.0		1.0
Burkina-Faso	2010	17,087		10.8		4.0
Burundi	2016–17	17,269	1.2	9.4	47.2	4.9
Cameroon	2011	15,426		32.3		0.8
Chad	2014–15	17,719		11.3		
Comoros	2012	5,329		38.4		4.3
Congo	2012	10,819		20.3		3.0
Cote d'Ivoire	2012	10,060		23.6		1.6
Democratic Republic of Congo	2013–14	18,827		14.5		4.0
Ethiopia	2016	15,683		11.7	32.9	1.4
Gabon	2012	8,422		38.6		3.0
Gambia	2013	10,233		22.6		0.3
Ghana	2014	9,396	5.2	35.6		0.5
Guinea	2012	9,142		18.9		
Kenya	2014	31,079	9.4	29.4	4.1	1.1
Lesotho	2014	6,621	17.3	44.5		8.4
Liberia	2013	9,239		24.6	23.7	1.1
Madagascar	2009	17,375		6.7		9.9
Malawi	2015-16	16,592		22.6		0.7
Mali	2013	10,424		19.3		1.1
Mozambique	2011	13,537		20.4		2.3
Namibia	2013	1,018		36.4	47.3	7.4
Niger	2012	11,160		20.2		2.7
Nigeria	2013	38,948		25.3		0.4
Rwanda	2014–15	13,497		23.2		2.1
Sao Tome and Principe	2008/09	2,615		34.3		1.6
Senegal	2011	15,688		18.4		0.5
Sierra Leone	2013	16,658		19.3		7.3
Tanzania	2015–16	13,266		28.5	13.7	0.9
Togo	2013–14	9,480		28.0		0.7
Uganda	2016	18,506		22.4		2.3
Zambia	2013–14	16,411		22.1	9.4	1.4
Zimbabwe	2015	9,955		37.3	13.0	0.5

The results showed that women aged 40–44 years and 45–49 years were least represented, respectively. Urban women accounted for 36.5% of respondents; while about two-third of women were Christians and have at most primary education. Approximately one-quarters (26.2%) of the women were currently never in union. The overall non-use of media was high specifically reading of newspaper or magazine (78.0%). The distribution of wealth-related quintile by maternal characteristics showed that 10% and 55.0% of women from urban and rural residence respectively, were below the middle class in wealth-related quintile. Furthermore, approximately 2.5, 19.7, 43.6, and 55.3% of women having higher, secondary, primary and no formal education respectively reported household wealth index below the middle class. While 28.3% of women were currently never in union reported below middle class in household wealth index, about 42.4% among ever married women accounted for same. About 44.7, 53.5, and 55.0% of non-users of newspaper or magazine, radio, and television respectively, reported below middle class in household wealth quintile. In addition, about 50% of women with high parity (≥4 children) reported below middle class in household wealth index. See Table 2 for details.

**Table 2:** Distribution of women's characteristics by wealth-related quintiles; Demographic and Health Surveys, 2008–2017.

**Variable**	***n***	**Poorest**	**Poorer**	**Middle**	**Richer**	**Richest**
**AGE**						
15–19	99,944 (21.2)	18,347 (18.4)	18,320 (18.3)	19,125 (19.1)	20,187 (20.2)	23,965 (24.0)
20–24	86,128 (18.3)	16,034 (18.6)	15,816 (18.4)	15,723 (18.3)	17,514 (20.3)	21,041 (24.4)
25–29	82,221 (17.5)	16,363 (19.9)	15,326 (18.6)	15,122 (18.4)	16,143 (19.6)	19,267 (23.4)
30–34	67,361 (14.3)	13,934 (20.7)	12,504 (18.6)	12,489 (18.5)	13,061 (19.4)	15,373 (22.8)
35–39	56,964 (12.1)	12,009 (21.1)	10,858 (19.1)	10,828 (19.0)	11,069 (19.4)	12,200 (21.4)
40–44	42,935 (9.1)	9,450 (22.0)	8,419 (19.6)	8,174 (19.0)	8,221 (19.1)	8,671 (20.2)
45–49	34,863 (7.4)	7,547 (21.6)	6,897 (19.8)	7,127 (20.4)	6,804 (19.5)	6,488 (18.6)
**PLACE OF RESIDENCE**						
Urban	171,897 (36.5)	7,539 (4.4)	9,954 (5.8)	20,346 (11.8)	43,922 (25.6)	90,136 (52.4)
Rural	299,361 (63.5)	86,323 (28.8)	78,347 (26.2)	68,387 (22.8)	49,268 (16.5)	17,036 (5.7)
**RELIGION**						
Christianity	292,827 (65.6)	52,167 (17.8)	53,002 (18.1)	55,648 (19.0)	59,811 (20.4)	72,199 (24.7)
Islam	127,841 (28.7)	27,886 (21.8)	25,065 (19.6)	24,551 (19.2)	24,924 (19.5)	25,415 (19.9)
Others/no religion	25,420 (5.7)	9,775 (38.5)	6,175 (24.3)	4,052 (15.9)	3,089 (12.2)	2,329 (9.2)
**LEVEL OF EDUCATION**						
No education	154,399 (32.8)	48,955 (31.7)	36,447 (23.6)	30,536 (19.8)	24,485 (15.9)	13,976 (9.1)
Primary	157,612 (33.4)	34,078 (21.6)	34,713 (22.0)	33,648 (21.3)	30,592 (19.4)	24,581 (15.6)
Secondary	139,247 (29.6)	10,691 (7.7)	16,771 (12.0)	23,486 (16.9)	34,814 (25.0)	53,485 (38.4)
Higher	19,945 (4.2)	135 (0.7)	356 (1.8)	1,054 (5.3)	3,289 (16.5)	15,111 (75.8)
**CURRENTLY WORKING**						
Yes	266,513 (58.7)	54,316 (20.4)	52,299 (19.6)	51,215 (19.2)	52,308 (19.6)	56,375 (21.2)
No	187,389 (41.3)	35,526 (19.0)	32,720 (17.5)	34219 (18.3)	37568 (20.0)	47,356 (25.3)
**CURRENT MARITAL STATUS**						
Never in union	127,102 (26.2)	17,017 (13.4)	18,911 (14.9)	22,824 (18.0)	27,592 (21.7)	40758 (32.1)
Currently in union/living with a man	315,211 (64.9)	69,749 (22.1)	64,592 (20.5)	61,332 (19.5)	59,880 (19.0)	59,658 (18.9)
Formerly in union/living with a man	43,323 (8.9)	10,010 (23.1)	8,165 (18.8)	7,988 (18.4)	8,244 (19.0)	8,916 (20.6)
**FREQUENCY OF READING NEWSPAPER/MAGAZINE**						
Not at all	367,100 (78.0)	86,798 (23.6)	77,375 (21.1)	73,158 (19.9)	69,032 (18.8)	60,737 (16.5)
Less than once a week	56,558 (12.0)	4,521 (8.0)	6,913 (12.2)	9,320 (16.5)	13,543 (23.9)	22,261 (39.4)
At least once a week	42,627 (9.1)	2,034 (4.8)	3,441 (8.1)	5,498 (12.9)	9,708 (22.8)	42,627 (51.5)
Almost everyday	4,080 (0.9)	301 (7.4)	415 (10.2)	548 (13.4)	741 (18.2)	2,075 (50.9)
**FREQUENCY OF LISTENING TO RADIO**						
Not at all	179,063 (38.0)	55,544 (31.0)	40,260 (22.5)	32,878 (18.4)	27,769 (15.5)	22,612 (12.6)
Less than once a week	93,281 (19.8)	16,414 (17.6)	17,807 (19.1)	18,321 (19.6)	19,136 (20.5)	21,603 (23.2)
At least once a week	176,116 (37.4)	19,763 (11.2)	26,961 (15.3)	33,281 (18.9)	41,039 (23.3)	55,072 (31.3)
Almost everyday	22,352 (4.7)	2,067 (9.2)	3,189 (14.3)	4,166 (18.6)	5,153 (23.1)	7,777 (34.8)
**FREQUENCY OF WATCHING TELEVISION**						
Not at all	272,469 (57.9)	81,128 (29.8)	68,537 (25.2)	59,148 (21.7)	45,369 (16.7)	18,287 (6.7)
Less than once a week	60,972 (13.0)	7,271 (11.9)	10,433 (17.1)	12,562 (20.6)	14,921 (24.5)	15,785 (25.9)
At least once a week	111,097 (23.6)	3,881 (3.5)	6,937 (6.2)	13,756 (12.4)	26,082 (23.5)	60,441 (54.4)
Almost everyday	25,915 (5.5)	1,411 (5.4)	2,245 (8.7)	3,111 (12.0)	6,648 (25.7)	12,500 (48.2)
**PARITY**						
Nil	124,666 (26.5)	16,943 (13.6)	18,669 (15.0)	21,809 (17.5)	26,476 (21.2)	40,769 (32.7)
1–3	179,046 (38.1)	33,391 (18.6)	32,604 (18.2)	32,713 (18.3)	36,293 (20.3)	44,047 (24.6)
≥4	166,702 (35.4)	43,350 (26.0)	36,867 (22.1)	34,066 (20.4)	30,230 (18.1)	22,189 (13.3)

**Figure 1:**
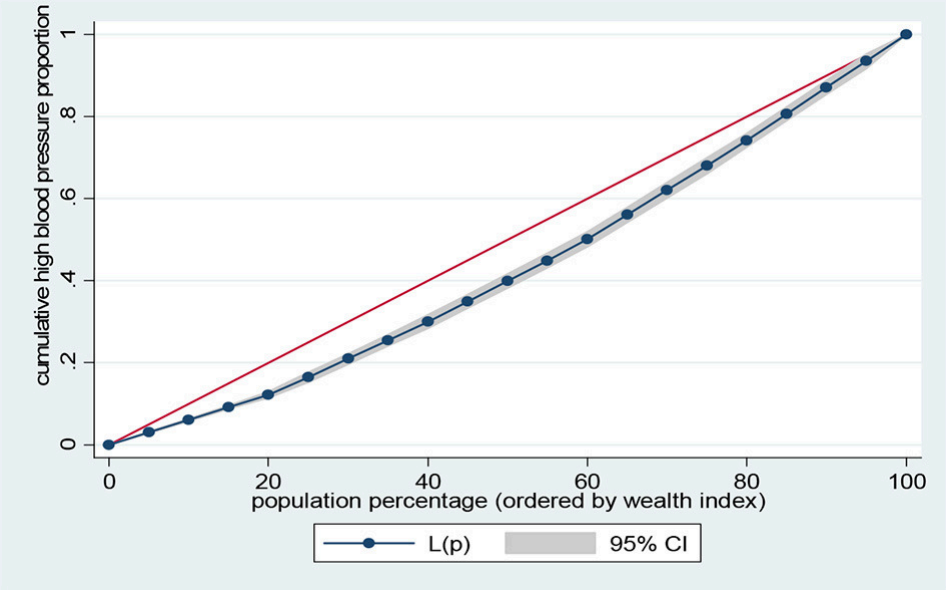
Lorenz curve for high blood pressure.

**Figure 2:**
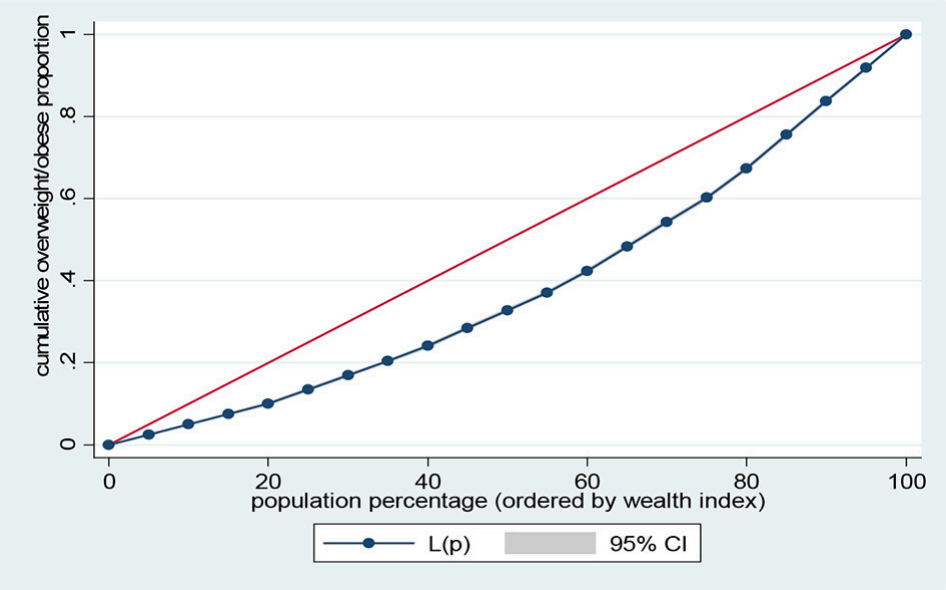
Lorenz curve for overweight/obesity.

**Figure 3:**
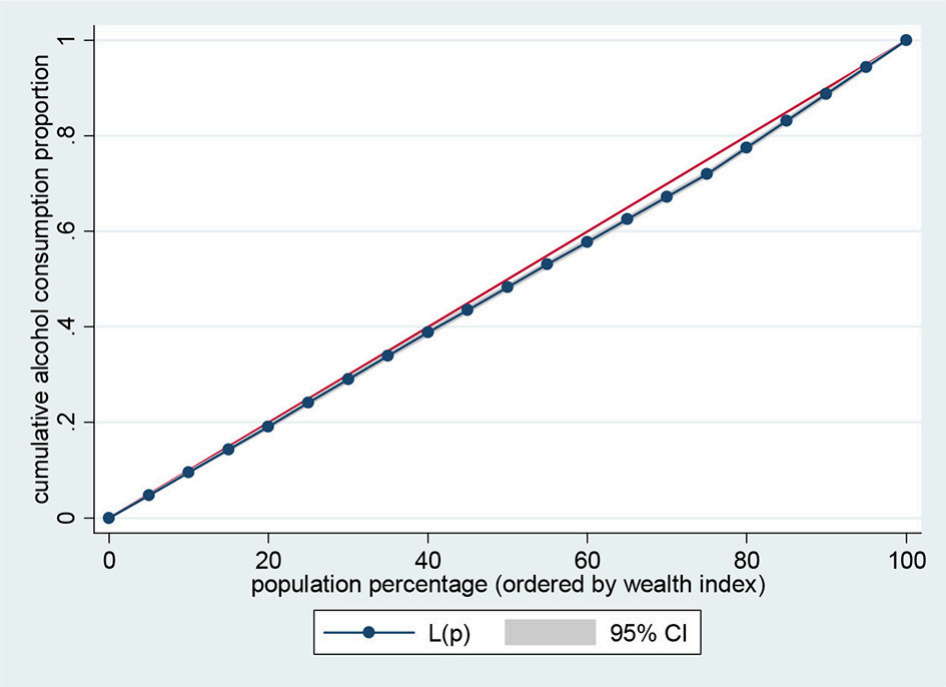
Lorenz curve for alcohol consumption.

**Figure 4:**
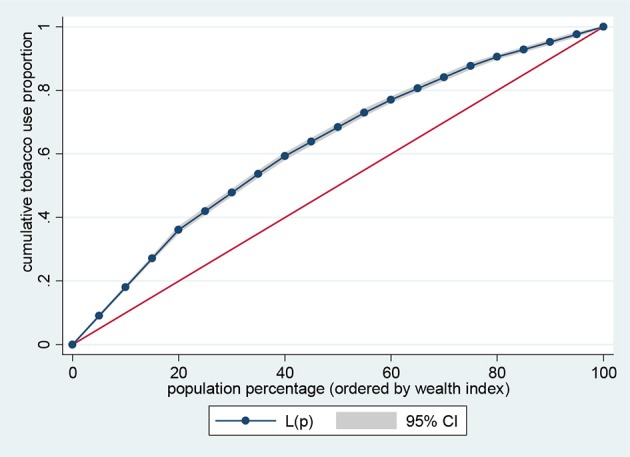
Lorenz curve for tobacco use.

Figures 1–4 graph the Lorenz curves for high blood pressure, overweight/obesity, alcohol consumption, and tobacco use. Lorenz curve is a graphical representation of the distribution of wealth (measured by household assets) across health outcomes ranked in increasing size of share. We used Lorenz curves to assess the total inequalities in these health indicators and their changes among women in sub-Saharan Africa countries by socioeconomic level. The Lorenz curves for high blood pressure, overweight/obesity and alcohol consumption were below the line of equality, indicating that the concentration is more among the higher socioeconomic group in these health indicators. However, the inequality was minimal in alcohol consumption as the areas between the curve and the line of inequality was greater for overweight/obesity, and high blood pressure than alcohol consumption. The more the Lorenz curves sags away from the line of equality, the greater the degree of inequality. Conversely, the Lorenz curve fell above the line of equality which implies that lower socioeconomic group has greater values of tobacco use than the higher socioeconomic group.

**Figure 5:**
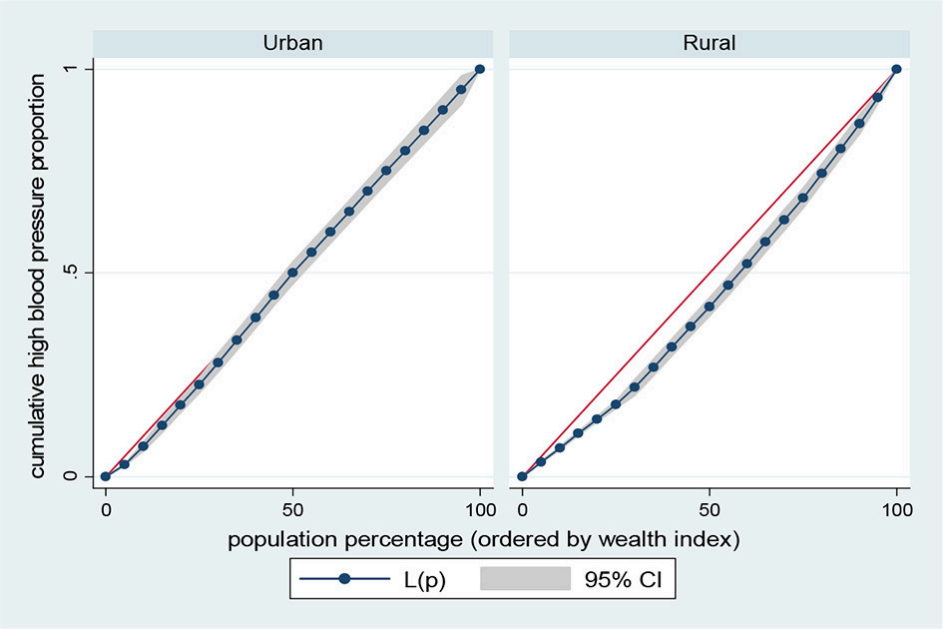
Urban-rural Lorenz curve for high blood pressure.

**Figure 6:**
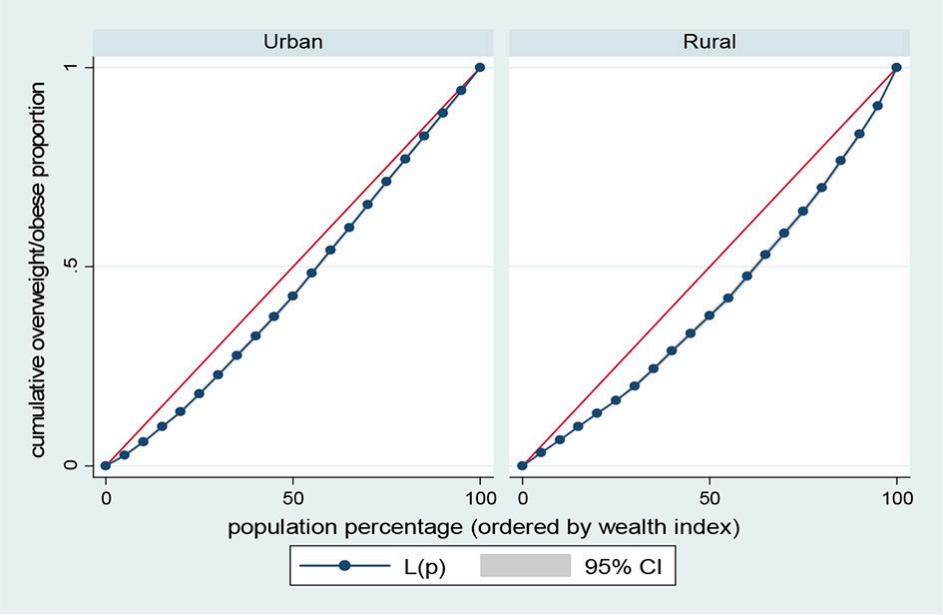
Urban-rural Lorenz curve for overweight/obesity.

**Figure 7:**
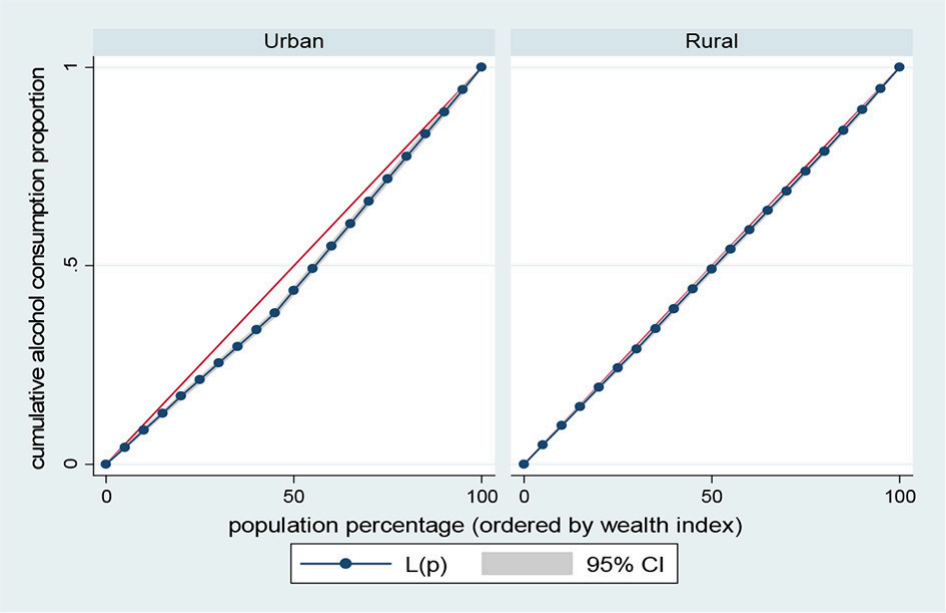
Urban-rural Lorenz curve for alcohol consumption.

**Figure 8:**
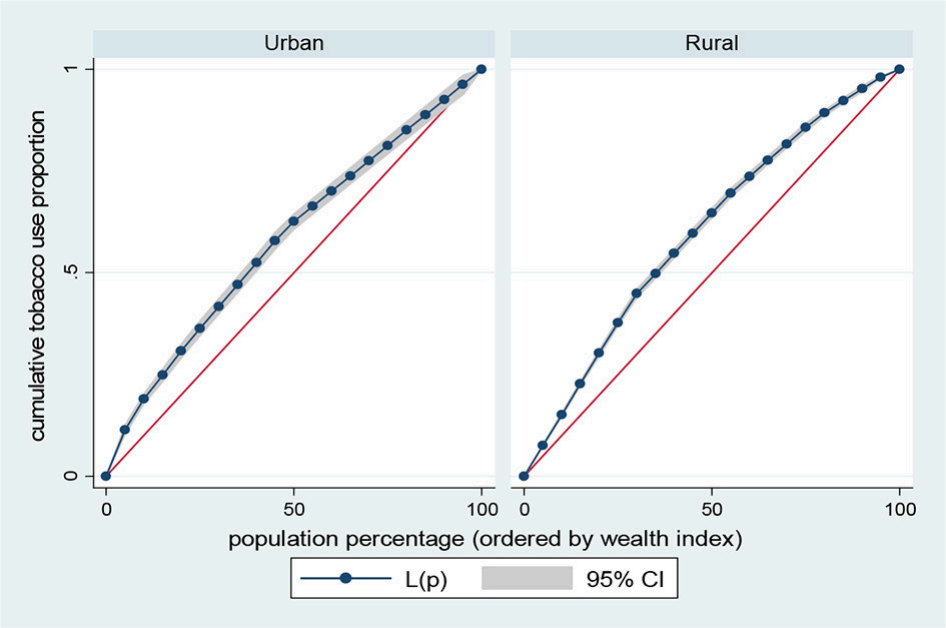
Urban-rural Lorenz curve for tobacco use.

Results presented in Figures 5–8 show the socioeconomic inequalities for behavioral and metabolic risk factors of NCDs by urban-rural place of residence. Figures 5, 6 indicate that rural women with high socioeconomic status had more high blood pressure and overweight/obesity than the urban women. Furthermore, Figure 7 shows that wealthy women from urban residence had higher alcohol consuption than the rural dwellers. The Lorenz curve presented in Figure 8 revealed that women of low socioeconomic group from urban residence have more tobacco use lifestyles. The more the Lorenz curves sags away from the line of equality, the greater the degree of inequality. The inequalities in socioeconomic level was less among urban women who have high blood pressure and among rural women who were involved in alcohol consumption; as the areas between the curve and the line of inequality was minimal. See Figures 5–8 for details.

The results of prevalence and concentration index (CI) values for high blood pressure, overweight/obesity, alcohol consumption, and tobacco use across socioeconomic quintiles are presented in Table 3. From the results, the overall prevalence of having high blood pressure was 5.5%, overweight/obesity accounted for about 23.1%, alcohol consumption was reported 23.9% of women and tobacco users was 2.4%, respectively. Further, rural vs. urban prevalence of high blood pressure, overweight/obesity, alcohol consumption, and tobacco use were reported by socioeconomic differentials. The concentration index, which was directly related to the curves, quantified the degree of socioeconomic related inequalities in high blood pressure (CI = 0.1352, *p* < 0.001); overweight/obesity (CI = 0.2285, *p* < 0.001) and alcohol consumption (CI = 0.0278, *p* < 0.001). These risk factors of NCDs were significantly more in the higher socioeconomic groups, compared to the lower socioeconomic groups. In contrast, the concentration index of tobacco use (CI = 0.2551, *p* < 0.001) was significantly more in lower socioeconomic group, compared to the higher socioeconomic group. The test for differences in rural vs. urban concentration indices for high blood pressure, overweight/obesity, alcohol consumption, and tobacco use were statistically significant in all the health indicators (*p* < 0.05). For the metabolic health indicators; rural residence had higher CI than their urban counterpart (high blood pressure: CI rural = 0.1156 vs. CI urban = 0.0173; *z* = 4.88; *p* < 0.001; overweight/obesity: CI rural = 0.1722 vs. CI urban = 0.0953; *z* = 20.94; *p* < 0.001). Conversely, for the behavioral health indicators; urban residence had higher CI than their rural counterpart (alcohol consumption: CI rural = 0.0158 vs. CI urban = 0.0689; *z* = −8.79; *p* < 0.001; tobacco use: CI rural = −0.2008 vs. CI urban = −0.1663; *z* = −2.87; *p* < 0.001). See Table 3 for details.

**Table 3:** Prevalence and concentration index (CI) of high blood pressure, overweight/obesity, alcohol consumption, and tobacco use by wealth quintiles; Demographic and Health Surveys, 2008–2017.

**Wealth index**	**High blood pressure**			**Overweight/obesity**			**Alcohol consumption**			**Tobacco use**		
	**Rural**	**Urban**	**Overall**	**Rural**	**Urban**	**Overall**	**Rural**	**Urban**	**Overall**	**Rural**	**Urban**	**Overall**
Poorest (%)	85.9	14.1	12.5	87.9	12.1	9.8	94.4	5.6	18.8	93.3	6.7	36.2
Poorer (%)	80.3	19.7	16.8	83.0	17.0	12.8	89.7	10.3	17.4	90.4	9.6	22.0
Middle (%)	70.8	29.2	19.3	70.3	29.7	16.3	79.6	20.4	17.5	80.5	19.5	17.2
Richer (%)	49.9	50.1	23.4	42.8	57.2	23.9	58.3	41.7	18.6	53.1	46.9	13.8
Richest (%)	19.2	80.8	28.1	13.1	86.9	37.1	15.5	84.5	27.6	16.1	83.9	10.8
Total (%)	54.9	45.1	5.5	45.8	54.2	23.1	62.5	37.5	23.9	76.6	23.4	2.4
Concentration index (CI)	0.1156	0.0173	0.1352	0.1722	0.0953	0.2285	0.0158	0.0689	0.0278	−0.2008	−0.1663	−0.2551
Standard error (SE)	0.0140	0.0145	0.0104	0.0029	0.0023	0.0019	0.0039	0.0046	0.0031	0.0060	0.0104	0.0053
P	<0.001	0.233	<0.001	<0.001	<0.001	<0.001	<0.001	<0.001	<0.001	<0.001	<0.001	<0.001
z-stat	4.88			20.94			−8.79			−2.87		
*p*-value	<0.001			<0.001			<0.001			0.004		

*z-stat, Test for statistically significant differences in rural vs. urban concentration index*.

## Discussion

In this study, we examined the socioeconomic inequalities of metabolic and behavioral risk factors of NCDs among women in sub-Saharan Africa countries. The findings include the current prevalence of NCDs risk factors such as high blood pressure, overweight/obesity, alcohol consumption, and tobacco use among women of reproductive age. The prevalence of high blood pressure was less than previous results among women in several African countries (11). The reduction could be due to health interventions that have been conducted over time. In addition, results were similar with the prevalence of overweight/obesity and alcohol consumption from previous studies (12, 17, 26). We notably found a low percentage of women who use tobacco products and the result is consistent with the report from a previous study (37). The low prevalence of tobacco use could be due to cultural sensitivity and religious barriers, such that in several religious beliefs and in African context, women's use of tobacco products is highly considered as improper behavior and remains highly unacceptable (38, 39). However, there could be more women who use tobacco products especially smokeless tobacco, but due to the fear of shame or ignorance may regard smoking as the only method of tobacco use and hence misreport their true status.

Findings about the direction and magnitude of socioeconomic inequalities revealed diverse patterns across metabolic and behavioral risk factors of NCDs. Tobacco use was significantly higher among lower socioeconomic women particularly among rural dwellers as presented (see the prevalence portion of Table 3). This is consistent with the reports from previous studies (4, 40). Conventionally, the practice of risky lifestyles such as tobacco use tends to shift from higher to lower socioeconomic groups. As society grows economically, Western lifestyles are adopted mostly by the well-off groups who, eventually move away from these practices based on the consequences or due to health interventions and the disadvantaged groups then adopt the lifestyles due to societal or environmental influence. This result points out the significance of integrating equity considerations in extensive tobacco use control interventions. Conversely, low socioeconomic women were found to have significantly less prevalence of high blood pressure, overweight/obesity, and alcohol consumption (see the prevalence portion of Table 3) than higher socioeconomic women, which is similar to previous reports (5, 41). Nonetheless, a systematic review involving several developing countries previously found overweight/obesity and alcohol consumption were significantly higher among lower socioeconomic women (4, 17). Generally, economic development could increase sedentary lifestyles or reduce physical activity levels. It could also lead to adoption of Western lifestyles which may contribute in dietary shift to improper food choices such as higher consumption of “fast foods” including sugar and fat which are probable factors for overweight/obesity. However, there could be fewer burdens of behavioral and metabolic risk factors of NCDs among the advantaged women through access to health information due to availability of resources.

Further, we found socioeconomic disparities in behavioral and metabolic risk factors for NCDs by rural or urban place of residence. The study revealed that high blood pressure and overweight/obesity were more prevalent among the higher socioeconomic women in rural areas. However, alcohol consumption was more among the higher socioeconomic women in urban areas than the rural dwellers. Conversely, women of lower socioeconomic status in urban areas had higher tobacco use. These findings are consistent with previous researchers who found the well-off groups or communities to have a lower risk of mortality from NCDs than the disadvantaged or hard-to-reach community dwellers (42). The WHO framework convention on tobacco control remarked on the level of burden the consumption of tobacco products place on disadvantaged groups (43). The socioeconomic inequalities across NCDs risk factors could be due to failure of past interventions to adopt equity-based strategies to terminate them because different populations may vary their levels of participation in health programmes or behavior change communication.

Based on our findings, the goal of reducing premature NCD mortality by a third by 2030 could be possible by leveraging development budgets to address the poverty-health bond in sub-Saharan Africa countries. Understanding the pattern and trend of socio-economic differentials in the risk factors for NCDs, could help improve NCD prevention, development, and poverty reduction strategies which are necessary in the global action plan for the prevention and control of NCDs (44). It is possible that development agencies working with the disadvantaged members of LMICs could readjust their programmes to address NCD by the clear evidence that NCDs risk factors affect these populations. The findings from this study bring to limelight the urgent need for disaggregated data emphasized at the United Nation High Level Meeting on NCDs (45). The socio-economic gradient of inequalities in the risk factors of NCDs could depend on the stage of economic development, cultural factors, social, and health policies. Women living in the remote or hard-to-reach communities and those with low socioeconomic status could have worse access to health care for prompt diagnosis and treatment of NCDs than those living in urban settings or those with higher socioeconomic status.

## Strengths and limitations

In this study, we used multi-national representative large data sets which provide generalizable estimates. Data were collected from numerous countries in sub-Saharan Africa which allowed large reporting of the prevalence of the risk factors of non-communicable diseases. However, using a cross-sectional study, we collected data across a number of countries at different points in time. Therefore, the distribution of NCDs behavioral and metabolic risk factors could have changed over time. Longitudinal studies would better track these changes over time and harmonize the drivers of these differences. Furthermore, we were unable to measure sources of unobserved heterogeneity across the dataset (46–48).

## Conclusion

We recognize that disparities in wealth distribution and the place of residence contribute to the burdens of NCDs risk factors. We conclude that high blood pressure and overweight/obesity were more prevalent among the well-off in rural areas. While alcohol consumption is more prevalent among the well-off women living in urban areas, however, tobacco use was more prevalent among women of low socioeconomic class in urban areas indicating a collaborative need for poverty-reduction oriented and location-based interventions. Specific attentions are necessary to address the risk factors of NCDs among these groups. Reduction in poverty level would contribute to the success of policies addressing factors that show wealth-based inequality, such as high blood pressure, overweight/obesity, alcohol consumption, and tobacco use among women of reproductive age. There is need to use behavior change communication to establish healthier lifestyles.

Furthermore, we recommend a comprehensive method to address NCDs risk factor reduction and health promotion, with efforts targeted to those at higher risk. A notable intervention would be to adopt a high-risk approach to reduce risk distribution by directing resources to high-risk women, who have higher likelihood of involving in unsafe lifestyles, consequently improving the benefit to risk-ratio and enhancing the cost effectiveness of preventive health programmes by highlighting the worth of equity-based strategies. Integrating equity elements to interventions is a key measure toward ensuring that policies and programmes aid high-risk women. Government should strengthen living standards, literacy and health care system to diminish the increasing prevalence of NCD risk factors. Effective actions to reduce NCDs risk factors could include; evidence-based strategies for alcohol and tobacco control, women's empowerment programmes especially in disadvantaged groups, development programmes and education, skilled care for delivery of preventive interventions and for early detection and treatment of NCDs risk factors, universal, cost-effective, and accessible health care system that target low socioeconomic status individuals.

## Data availability statement

Data for this study were sourced from Demographic and Health surveys (DHS) and available from: https://www.dhsprogram.com/data/available-datasets.cfm

## Ethics statement

Ethics approval for this study was not required since the data is secondary and is available in the public domain. More details regarding DHS data and ethical standards are available at: http://goo.gl/ny8T6X.

## Author contributions

SY and ME contributed to the study design, the review of literature, and analysis of literature, manuscript conceptualization, and preparation. OU and GB critically reviewed the manuscript for its intellectual content. SY had final responsibility to submit for publication. All authors read and approved the final manuscript.

### Conflict of interest statement

The authors declare that the research was conducted in the absence of any commercial or financial relationships that could be construed as a potential conflict of interest.

## Abbreviations

CI: Concentration Index
DHS: Demographic Health Survey
NCDs: Non-communicable diseases
PCI: Principal components analysis
SDGs: Sustainable Development Goals
SSA: Sub-Saharan Africa.
